# 2528. Cefepime Induced Neurotoxicity in Patients With or Without a History of Seizures

**DOI:** 10.1093/ofid/ofad500.2146

**Published:** 2023-11-27

**Authors:** Nadeem Baalbaki, Christopher Hoge, Aleena Dar, Mahinaz Mohsen, Joachim Sackey, Tanzila Salim

**Affiliations:** University Hospital, Newark, New Jersey; Rutgers NJMS, Newark, New Jersey; Rutgers New Jersey Medical School, Newark, New Jersey; Rutgers New Jersey Medical School, Newark, New Jersey; Rutgers School of Public Health, Newark, NJ; NJMS RUTGERS, Newark, New Jersey

## Abstract

**Background:**

Cefepime is a commonly utilized antimicrobial for the treatment of nosocomial infections and serves as a carbapenem sparing agent for the treatment of ampC inducible bacteria. Although generally tolerated, cefepime induced neurotoxicity (CIN) is a well-documented adverse effect that has been observed most significantly in patients with renal dysfunction and elevated serum troughs. Data describing the incidence of CIN in patients with a history of seizures is currently limited.

**Methods:**

This was a retrospective matched cohort study of patients admitted to University Hospital from May 2020 to May 2022 who were treated with cefepime for > 48 hours with or without a baseline history of seizures. Patients with seizures were identified using ICD-10 codes. Patients were matched at a rate of 1:1 by age (+/- 5), sex at birth, and month of admission (+/- 1). The primary outcome was the rate of CIN in patients with a history of seizures versus patients without a history of seizures. The secondary outcome was identification of risk factors associated with CIN.

**Results:**

A total of 102 patients were included in this study (51 in each group). There was no statistically significant difference in the rate of CIN in patients with a history of seizures versus patients without a history of seizures (11.8% vs 5.9%, P = 0.487). In a univariate analysis, patients with acute kidney injury (OR, 13.1 [95% CI, 2.3-74.8]) and an intensive care unit (ICU) stay (OR, 5.3 [95% CI, 1.04-36.4]) were associated with an increased risk of CIN. In a multivariate analysis, duration of cefepime administration was the only risk factor associated with CIN that was statistically significant (OR, 1.94 [95% CI 1-3.8]).

**Conclusion:**

There was no statistically significant difference in the rate of CIN in patients with a history of seizures compared to patients without a history of seizures, although there were twice as many patients with CIN in the seizure group. Patients with acute kidney injury and an ICU stay were more likely to experience CIN in a univariate analysis, however confidence intervals were wide requiring caution with interpretation. There was a 1.94 times increase in the odds of CIN with every additional day of cefepime administration. Further investigations with larger sample sizes are warranted to confirm these findings.

**Disclosures:**

**All Authors**: No reported disclosures

